# Multicenter Prospective Cohort Study of the Patient-Reported Outcome Measures PRO-CTCAE and CAT EORTC QLQ-C30 in Major Abdominal Cancer Surgery (PATRONUS): A Student-Initiated German Medical Audit (SIGMA) Study

**DOI:** 10.1245/s10434-021-09646-z

**Published:** 2021-03-08

**Authors:** CHIR-Net SIGMA Study Group*, André Mihaljevic, Christopher Fink, Pia Frey, Mirco Friedrich, Alexander Leuck, Lukas Rädeker, Marius Schwab, Colette Doerr-Harim, Solveig Tenckhoff, Manuel Feißt, Sara Fatima Faqar-Uz-Zaman, Niall Brindl, Thomas Jing Zhi Tu, Alexander Studier-Fischer, Charlotte Kuner, Julia Gsenger, Clara Möhwald, Markus Prem, Henriette Von der Waydbrink, Katharina Tielking, Mohammad Rahbari, Boortz Bertram, Jens Neudecker, Linda Jörke, Lisa Findeisen, Pascal Fichtel, Richard Bigge, Rahbari Nuh, Ulrich Bork, Adele Klein, Strahin Stojanovich, Andreas A. Schnitzbauer, Samuel Vorbach, Jan Braune, Alexander Scholz, Paul Kreifels, Daniel Zell, Tim Van de Lo, Philipp Holzner, Verena Martini, Nico Hinz, Anna Ekaterina Finger, Nadia Kokaly, Ricardo Kosch, Sharlaine Yun Lan Piel, Michael Tachezy, Eva Gablenz, Obada T. Alhalabi, Svenja Sliwinski, Alexander Marx, Annette AuYeung, Christina Achilles, Fynn Betge, Jana Kühn, Karam Al Halabi, Lennart von Fritsch-Seerhausen, Lydia Beck, Marc Truant, Sophie Preuß, Stella Waider, Till Seiboldt, Anja Sander, Hannah Honecker, Svenja Seide, Dorothea Weber, Christopher Büsch, Florian Wagner, Lisa Reckmann, Anna-Maria Sonnenberg, Michael Zaczeck, Joscha Buech, Laura Luden, Marie-Pauline Autenrieb, Terbish Taivankhuu, Desiree Tröster, Simon Engster, Alexander Buia, Ernst Hanisch, Charlotte Müller-Debus, Alexander Heinrich, Hannah Rasel, Kartal Nergiz, Hauke Lang, Felix Watzka, Laura Pohl, Akiko Lucia Makabe, Anna-Maria Seckler, Blerina Misiraj, Carmel Kerem, Christian Lull, Jonas Voigt, Julian Pascal Beier, Leon Schütze, Leonhard Dreyer, Marie Therese Kleinsorge, Thao Nguyen, Thomas Thäwel, Tobias Günther, Florian Herrle, Diana Sadeghi, Anne Scheuerpflug, Antonia Apfelbeck, Sebastian Schömer Cuenca, Markus Albertsmeier, Petra Zimmermann, Viktor von Ehrlich-Treuenstätt, Alessia Kopp, Lena Schmitzer, Daniel Reim, Rebekka Schirren, André L. Mihaljevic

**Affiliations:** 1 CHIR-Net SIGMA Coordination Centre, Heidelberg, Germany; 2 Department of General, Visceral and Transplantation Surgery, Heidelberg University Hospital, Heidelberg, Germany

## Abstract

**Background:**

The patient-reported outcomes (PRO) version of the Common Terminology Criteria for Adverse Events (PRO-CTCAE) and the computerized adaptive testing (CAT) version of the EORTC quality-of-life questionnaire QLQ-C30 have been proposed as new PRO measures in oncology; however, their implementation in patients undergoing cancer surgery has not yet been evaluated.

**Methods:**

Patients undergoing elective abdominal cancer surgery were enrolled in a prospective multicenter study, and postoperative complications were recorded according to the Dindo–Clavien classification. Patients reported PRO data using the CAT EORTC QLQ-C30 and the PRO-CTCAE to measure 12 core cancer symptoms. Patients were followed-up for 6 months postoperatively. The study was carried out by medical students of the CHIR-*Net* SIGMA study network.

**Results:**

Data of 303 patients were obtained and analyzed across 15 sites. PRO-CTCAE symptoms ‘poor appetite’, ‘fatigue’, ‘exhaustion’ and ‘sleeping problems’ increased after surgery and climaxed 10–30 days postoperatively. At 3–6 months postoperatively, no PRO-CTCAE symptom differed significantly to baseline. Patients reported higher ‘social functioning’ (*p* = 0.021) and overall quality-of-life scores (*p* < 0.05) 6 months after cancer surgery compared with the baseline level. There was a lack of correlation between postoperative complications or death and any of the PRO items evaluated. Feasibility endpoints for student-led research were met.

**Conclusion:**

The two novel PRO questionnaires were successfully applied in surgical oncology. Postoperative complications do not affect health-reported quality-of-life or common cancer symptoms following major cancer surgery. The feasibility of student-led multicenter clinical research was demonstrated, but might be enhanced by improved student training.

**Supplementary Information:**

The online version contains supplementary material available at 10.1245/s10434-021-09646-z.

Cancer is the second leading cause of death in Germany, Europe, and the US.^1^–^4^ In 2018, there were an estimated 1.7 million and 4 million new cancer cases in the US and Europe, respectively.^3^,^4^ A broad range of outcome parameters is available to evaluate the risks and benefits of oncological treatments and their effect on the personal well-being of patients. While survival can be considered an important efficacy endpoint in oncological studies,^5^,^6^ the overall value or benefit of ‘survival’ as judged by cancer patients might vary significantly depending on the clinical setting (palliative vs. curative), personal beliefs, physical and mental health, and other factors. In order to capture the “personal assessment of the burden and impact of a malignant disease and its treatment”,^7^ patient-reported outcome (PRO) measures have been developed, defined as “… outcomes collected directly from the patient without interpretation by clinicians or others”.^8^ With patients growing older and increasingly comorbid, the implementation of PRO measures helps to complement efficacy endpoints such as survival or morbidity and safety data, thus adding the patients’ perspective to clinical trials.^9^ The application of PRO measures in surgical oncology is of special interest not only as a clinical outcome parameter in routine care and clinical trials, but it might also be used to manage symptoms and complications.^10^

Thousands of PRO measures have been developed in medicine,^11^ however, in cancer patients, health-reported quality-of-life (HRQoL) and symptom scores are arguably the most important PROs.^12^ Contrary to symptom measures, HRQoL is a multidimensional tool “… encompassing physical and occupational function, psychological state, social interaction and somatic sensation”.^13^ HRQoL and symptom PRO measures can be categorized into generic, cancer-specific, or disease-specific questionnaires.^12^ Generic measures allow the comparison with healthy individuals, while cancer- and disease-type-specific tools aim to measure symptoms and HRQoL in all cancer patients or patients with a specific cancer disease, respectively.

A National Cancer Institute (NCI) consensus conference has proposed 12 cancer-specific symptoms that should be evaluated in all cancer trials (i.e. cancer-specific), but has left open which PRO measures should be used.^14^ The recently developed PRO version of the Common Terminology Criteria for Adverse Events (PRO-CTCAE™) could be a possible candidate but no data from patients undergoing abdominal cancer surgery have yet been published.^15^ Therefore, the feasibility and absolute values of PRO-CTCAE™ assessment remain unclear in this population. Similarly, the European Organisation for Research and Treatment of Cancer (EORTC) has recently developed a computerized adaptive testing (CAT) version of their HRQoL questionnaire QLQ-C30 (CAT EORTC QLQ-C30).^16^ The CAT EORTC QLQ-C30 is a cancer-specific questionnaire, i.e. it allows the comparison of HRQoL across multiple cancer types. Again, no data have yet been published for patients undergoing abdominal cancer surgery for this tool. In addition, understanding whether PROs overlap with or diverge from clinical outcomes such as survival and postoperative complications is critical to their application in quality assessment and improvement strategies. Therefore, the objectives of this cohort study were as follows.

1. Describe the absolute values of (a) CAT EORTC QLQ-C30 and (b) a set of 12 cancer-specific symptoms^14^ measured via the PRO-CTCAE™^15^ for patients undergoing major abdominal cancer surgery.
2. Describe the relationship between short-term clinical outcomes (morbidity) and PRO-CTCAE™ measurements in the short-term (within 30 postoperative days [POD]) and long-term (after 3 and 6 months).
3. Correlate short-term clinical outcomes (morbidity) with the long-term HRQoL at 3 and 6 months.
4. Describe the relationship between long-term clinical outcome (overall survival) with PRO-CTCAE™ and HRQoL according to the CAT version of the EORTC QLQ C-30.

The PATRONUS study was conducted by medical students under the supervision of academic surgeons (CHIR-*Net* SIGMA study group; see the Methods section). Student-led multicenter clinical research is a relatively novel field, therefore it is unclear how well medical students can motivate patients to submit PRO data and whether a large group of students remain dedicated to accomplish a multicenter prospective study. Hence, the following two additional educational objectives were evaluated.

1. To evaluate the rate of missing PRO data in a student-led research network.
2. To train and interest medical students for clinical research and surgery, defined as number of participating trial sites including patients compared with initiated trial sites (feasibility endpoint).

## Methods

### Study Design

PATRONUS is a multicenter, prospective, single-arm, observational cohort study conducted according to the published study protocol.^17^ The study report was written according to the current STROBE cohort guidelines.^18^

### Setting

The PATRONUS study has been initiated, conducted, analyzed, and reported by the Student-Initiated German Medical Audit (SIGMA) study group. SIGMA is a Germany-wide, student-led clinical research network affiliated to the CHIR-*Net*, the clinical trial network of the German Surgical Society.^19^ SIGMA offers medical students the opportunity to participate in student-led clinical research under the supervision of academic surgeons. Participating medical students are trained in workshops to acquire the theoretical and practical know-how to conduct substantial parts of clinical studies independently and act as peer-teachers for fellow students.^20^ Data analyses, interpretation, and reporting are performed by student members under the auspices of statisticians and CHIR-*Net* facilitators. PATRONUS is the first clinical study of the SIGMA study group.^17^

The study was conducted at the following sites: University Hospitals of Heidelberg, Berlin, Dresden, Frankfurt, Freiburg, Hamburg, Kiel, Lübeck, Mannheim, Münster, Mainz, Munich (Ludwig-Maximilians University and Technische Universität München), and two non-university academic hospitals—Evangelical Hospital Herne and Asklepios Hospital Langen. The PATRONUS study was approved by the responsible independent Ethics Committees (Heidelberg: 11th September 2017, reference S-466/2017) and was registered with the German Clinical Trials Register (DRKS00013035) on 26 October 2017. Patients were recruited between February 2018 and March 2019 and were followed-up until 6 months after surgery.^17^

Patient data were obtained using electronic case report forms (eCRF) entered into the REDCap electronic data capture system.^21^ Data security was assured by restricting data access to authorized and trained study members only. Based on a study-specific data validation plan, queries were created in case of missing data or implausible data entry, which had to be clarified by the study investigators and medical students to enhance the validity of data collection. Data were either obtained from patients or their records, or entered directly by patients themselves (for PRO measures)

### Participants

Adult (≥18 years) patients were screened preoperatively. Patients scheduled for elective abdominal surgery for confirmed or suspected malignancy were approached for informed consent. Inclusion criteria were^17^ (1) patient age ≥18 years; (2) patient was scheduled for elective abdominal surgery for confirmed or suspected malignancy; (3) patient’s ability to understand the character of the study; (4) planned laparoscopic or open surgery or any variant (i.e. laparoscopic-assisted, laparoscopic-thoracoscopic); and (5) written informed consent. Exclusion criteria were (1) language barrier that impedes follow-up or informed consent; and (2) American Society of Anesthesiologists (ASA) grade 4 or higher.

Demographic and baseline data, as well as a first set of PRO measures, were gathered during visit 1 (screening visit). Surgical data were collected in visit 2 (surgery), followed by short- and long-term clinical outcomes (postoperative complications and PRO measures) in visits 3 (postoperative day (POD) 3–5), 4 (POD 6–8), 5 (POD 10–14), 6 (POD 30 or at discharge) and 7 and 8 (3 and 6 months postoperatively, respectively) [electronic supplementary Fig. 1].

### Variables and Data Sources/Measurement

Both PROs and clinical outcomes were gathered.

1. The German translation of the PRO-CTCAE™^15^,^22^ was used to assess a core set of 12 cancer-associated symptoms as recommended by the NCI (fatigue, insomnia, pain, anorexia, dyspnea, cognitive problems, anxiety, nausea, depression, sensory neuropathy, constipation, and diarrhea).^14^
2. The CAT version of the quality-of-life questionnaire EORTC-QLQ 30 was used to evaluate HRQoL,^23^ and comprises five functional scales (physical, role, cognitive, emotional, and social functioning), three symptom scales (fatigue, pain, and nausea and vomiting), and a global health and quality-of-life scale. The CAT EORTC QLQ-C30 questionnaire takes patients’ individual priorities into account to increase precision.^16^ An online survey tool of the EORTC CAT group was used that was linked to the REDCap study database.^21^
3. Postoperative complication grades II–V within 30 days according to the Dindo–Clavien classification (DCC)^24^ (short-term clinical outcome). Complications were defined as minor (grade II) and major (grade III–V).
4. Long-term clinical outcome, defined as overall survival within 6 months postoperatively.

### Sample Size

This was a cohort study with an explorative nature, thus no formal sample size calculation was performed. The initial goal was to achieve an average recruitment rate of 30 patients per center.^17^

### Statistical Methods

All evaluations were carried out using the SAS version 9.4 software package (SAS Institute Inc., Cary, NC, USA). Being an exploratory study, the analysis is descriptive, and all *p*-values have to be interpreted in a descriptive sense. *p*-values <0.05 were determined as significant (in a descriptive sense). Missing values were described by relative and absolute frequencies, but were not imputed and were therefore dropped in the respective analyses. The number of recruited patients was smaller than planned,^17^ which is why most of the analyses were only performed as univariate analyses. For the analysis of the correlation between PRO and short- and long-term clinical outcome, group comparisons of the subscales of CAT EORTC QLQ-C30 and PRO CTCAE were performed by analysis of variance and Chi-square tests. Continuous variables were described using several non-missing values, mean, standard deviation, median, Q1, Q3, minimum and maximum. Moreover, for continuous variables, variance analyses between subgroups divided to the tumor entities (upper gastrointestinal, pancreatic, hepatobiliary, colorectal, others) and t-tests for all pairwise comparisons of these entities were performed. For binary or categorical variables, absolute and relative frequencies were provided. In addition, Chi-square tests were calculated for binary or categorical variables between subgroups divided into the tumor entities. All objectives were analyzed for every visit where the required data were collected (visits 3–8). PRO-CTCAE™ scores and EORTC QLQ-C30 scores were adjusted for baseline (visit 1), i.e. for correlation analyses the differences between visit and baseline values were calculated. For regression analyses, baseline values were complemented into the analyses as an independent variable, and for the final analysis, missing values in the items of PRO-CTCAE™ and CAT EORTC QLQ-C30 were handled as described in the scoring manuals of the two QoL measures. Further missing values were documented, and frequencies were described with descriptive methods. Since there is no official scoring method for PRO-CTCAE™, the mean of the item values for each of the 12 symptoms was used for the analysis. If all values for a subscale are missing, the mean of this subscale will also be set to missing. In addition, Kaplan–Meier graphs and log-rank tests between different tumor entities were performed regarding overall survival. Pairwise log-rank tests were performed using the ‘tukey’ method for adjustment for multiple testing. Cox regression analysis to evaluate possible relationships of time to death regarding overall survival and different baseline covariates were performed as univariate analyses due to the small number of events. Spearman’s rank correlations between short-term clinical outcomes (Comprehensive Complication Index [CCI] value)^25^ and the set of PRO-CTCAE™ (difference between visit and baseline score) in the short- and long-term were performed, and the long-term HRQoL PRO measure (difference between visit and baseline score) was analyzed for all subscales. Correlation strengths were defined as 0.00–0.19, ‘very weak’; 0.20–0.39, ‘weak’; 0.40–0.59, ‘moderate’, 0.60–0.79, ‘strong’, and 0.80–1.0 ‘very strong’. The entire statistical analysis was predetermined in a statistical analysis plan (SAP) written before closure of the database.

### Data Sharing

Requests for data sharing will be reviewed on an individual basis by the Steering Committee or the coordinating investigator. The data sharing process will comply with the good practice principles for sharing individual participant data, and data sharing will be undertaken in accordance with the required regulatory requirements. In particular, the privacy of the patients (i.e. sharing of anonymous data only) will be followed throughout.

## Results

### Participants

**Fig. 1:**
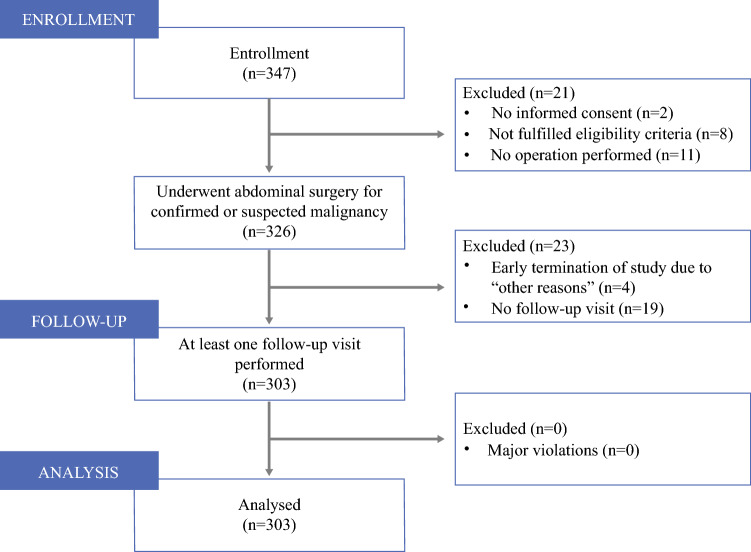
Study selection process. Recruitment took place between February 2018 and March 2019, with a total of 347 patients being enrolled, of whom 21 were excluded prior to surgery and another 23 were excluded postoperatively. No patients were excluded due to major protocol violations during follow-up

From February 2018 to March 2019, 347 patients were enrolled in the study at 15 German centers (Fig. 1). A total of 21 patients were excluded for the following reasons: 11 patients did not undergo surgery, 8 patients were ineligible, and 2 patients retracted their informed consent. The remaining 326 patients underwent surgery for confirmed or suspected malignancy. A total of 23 patients were either lost to follow-up after enrollment (*n* = 19) or terminated the study earlier due to other reasons (*n* = 4). Thus, 303 patients (87%) of the 347 enrolled patients were considered for subsequent analysis (Fig. 1).

### Baseline Data

The most frequent cancer type was hepatobiliary (*n* = 85, 28.05%), while the least frequent cancer type was upper gastrointestinal malignancies (*n* = 48, 15.8%) (Table 1). Participants undergoing pancreatic cancer surgery were significantly older than patients with hepatobiliary tumors, with the latter having more ASA III scores. Weight loss was frequent, especially in patients with upper gastrointestinal and pancreatic tumors (68.75% and 56.92%, respectively). Only 15% of pancreatic cancer patients had received neoadjuvant treatment, while almost half of the upper gastrointestinal patients had undergone neoadjuvant therapy (47.92%). BMI and medical comorbidities as common risk factors for postoperative complications did not differ significantly between tumor entities. Medical comorbidities were frequent (82.84%).

**Table 1:** Baseline data

	Upper GI [*n *= 48]	Pancreatic [*n *= 65]	Hepatobiliary [*n *= 85]	Colorectal [*n *= 65]	Other [*n *= 40]	Total [*n *= 303]	*p*-value
Age, years							
Mean ± SD	63.8 ± 11.7	65.8 ± 11.9	60.7 ± 12.6	63.8 ± 11.0	55.8 ± 13.5	62.3 ± 12.4	<0.001^a2^
BMI							
Mean ± SD	26.4 ± 4.8	25.2 ± 5.8	26.6 ± 4.2	26.3 ± 5.0	25.6 ± 5.4	26.1 ± 5.0	0.477^a2^
Sex							
Male	38 (79.17)	31 (47.69)	52 (61.18)	39 (60.00)	20 (50.00)	180 (59.41)	
Female	10 (20.83)	34 (52.31)	33 (38.82)	26 (40.00)	20 (50.00)	123 (40.59)	0.011^a1^
ASA status							
I	1 (2.08)	0 (0.00)	4 (4.76)	2 (3.08)	2 (5.26)	9 (3.00)	
II	20 (41.67)	41 (63.08)	28 (33.33)	38 (58.46)	22 (57.89)	149 (49.67)	0.008^a1^
III	27 (56.25)	24 (36.92)	52 (61.90)	25 (38.46)	14 (36.84)	142 (47.33)	
Missing	0	0	1	0	2	3	
Weight loss^b^							
No	15 (31.25)	28 (43.08)	51 (60.00)	37 (57.81)	22 (55.00)	153 (50.66)	
Yes	33 (68.75)	37 (56.92)	34 (40.00)	27 (42.19)	18 (45.00)	149 (49.34)	0.010^a1^
Missing	0	0	0	1	0	1	
Smoking							
Active-smoker	5 (10.42)	13 (20.00)	12 (14.12)	15 (23.08)	9 (22.50)	54 (17.82)	
Former smoker	22 (45.83)	14 (21.54)	27 (31.76)	21 (32.31)	16 (40.00)	100 (33.00)	0.124^a1^
Non-smoker	21 (43.75)	38 (58.46)	46 (54.12)	29 (44.62)	15 (37.50)	149 (49.17)	
Alcohol consumption							
Yes, often (almost daily)	9 (18.75)	7 (10.77)	6 (7.06)	12 (18.46)	8 (20.00)	42 (13.86)	
Yes, approximately once a week	16 (33.33)	14 (21.54)	24 (28.24)	13 (20.00)	9 (22.50)	76 (25.08)	0.429^a1^
Yes, less than once a week	14 (29.17)	22 (33.85)	28 (32.94)	19 (29.23)	13 (32.50)	96 (31.68)	
No	9 (18.75)	22 (33.85)	27 (31.76)	21 (32.31)	10 (25.00)	89 (29.37)	
Cholestasis							
No	43 (89.58)	49 (75.38)	69 (81.18)	61 (93.85)	38 (95.00)	260 (85.81)	
Yes	5 (10.42)	16 (24.62)	16 (18.82)	4 (6.15)	2 (5.00)	43 (14.19)	0.007^a1^
Prior abdominal surgeries							
No	28 (58.33)	32 (49.23)	20 (23.53)	29 (44.62)	11 (27.50)	120 (39.60)	
Yes	20 (41.67)	33 (50.77)	65 (76.47)	36 (55.38)	29 (72.50)	183 (60.40)	<0.001^a1^
Neoadjuvant treatment							
No	25 (52.08)	55 (84.62)	49 (57.65)	44 (67.69)	28 (70.00)	201 (66.34)	
Yes	23 (47.92)	10 (15.38)	36 (42.35)	21 (32.31)	12 (30.00)	102 (33.66)	0.002^a1^
Current medication							
No	7 (14.58)	8 (12.50)	12 (14.12)	16 (24.62)	8 (20.00)	51 (16.89)	
Yes	41 (85.42)	56 (87.50)	73 (85.88)	49 (75.38)	32 (80.00)	251 (83.11)	0.335^a1^
Medical comorbidities							
No	11 (22.92)	10 (15.38)	10 (11.76)	12 (18.46)	9 (22.50)	52 (17.16)	
Yes	37 (77.08)	55 (84.62)	75 (88.24)	53 (81.54)	31 (77.50)	251 (82.84)	0.422^a1^
If yes, cardiovascular	29 (61.70)	38 (59.38)	55 (64.71)	32 (49.23)	17 (42.50)	171 (56.81)	0.105^a1^
If yes, pulmonary	9 (19.15)	10 (15.63)	15 (17.65)	8 (12.50)	4 (10.00)	46 (15.33)	0.707^a1^
If yes, endocrine	11 (23.40)	28 (43.75)	29 (34.12)	13 (20.31)	12 (30.00)	93 (31.00)	0.041^a1^
If yes, gastrointestinal	8 (17.02)	15 (23.44)	20 (23.53)	12 (18.75)	11 (27.50)	66 (22.00)	0.741^a1^
If yes, musculoskeletal	14 (29.79)	18 (28.13)	14 (16.47)	12 (18.75)	7 (17.50)	65 (21.67)	0.241^a1^

Data are expressed as *n* (%) unless otherwise specified

*ASA* American Society of Anesthesiologists, *GI* gastrointestinal, *ANOVA* analysis of variance, *BMI* body mass index, *SD* standard deviation

^a^1 = Chi-square test, two-sided; 2 = ANOVA, two-sided

^b^Weight loss was recorded for the 6 months before surgery

### Surgery

The duration of operations varied between entities, ranging from 212.0 ± 113.0 min for hepatobiliary surgeries to 301.2 ± 134.2 min for pancreatic surgeries (Table 2). The estimated blood loss was more than twice as high in pancreatic surgery than in colorectal surgery (748 ± 708 mL vs. 353.8 ± 502.7 mL). In over 95% of cases, colorectal cancers were resected, while only 80% of hepatobiliary tumors were resectable. All patients undergoing upper gastrointestinal surgery had malignant tumors in their final histology, whereas 23.44% of pancreatic patients exhibited benign neoplasms in their final work-up. Overall, most patients had either pT2 (21.88%) or pT3 (28.47%) tumors. Significantly more patients undergoing colorectal surgery had R0 resections (93.18%) compared with pancreatic surgeries (54.10%) or other cancer surgeries (37.50%). The most frequent histological tumor types in the ‘other’ cancer cohort were adenocarcinomas (43.59%) and sarcomas (12.82%).

**Table 2:** Surgery data

	Upper GI	Pancreatic	Hepatobiliary	Colorectal	Other	Total	*p*-value
Duration of surgery, min							
*N*	48	65	85	65	39	302	<0.001^a2^
Mean ± SD	276.6 ± 126.0	301.2 ± 134.2	212.0 ± 113.0	264.6 ± 104.6	245.7 ± 168.0	257.2 ± 129.8	
Estimated blood loss, mL							
*N*	41	59	74	60	38	272	0.006^a2^
Mean ± SD	310.2 ± 244.9	748.1 ± 708.3	588.8 ± 671.8	353.8 ± 502.7	634.2 ± 1177.8	535.9 ± 713.6	
No tumor resection performed	4 (8.33)	6 (9.23)	17 (20.24)	3 (4.62)	6 (15.38)	36 (11.96)	0.036^a1^
Intraoperative blood transfusion							
Yes	3 (6.25)	11 (16.92)	6 (7.14)	5 (7.94)	9 (22.50)	34 (11.33)	0.034^a1^
Tumor type							
Adenocarcinoma	38 (79.17)	40 (61.54)	41 (48.24)	56 (86.15)	17 (43.59)	192 (63.58)	<0.001^a1^
Squamous cell carcinoma	4 (8.33)	0 (0.00)	3 (3.53)	2 (3.08)	0 (0.00)	9 (2.98)	
Neuroendocrine tumor	0 (0.00)	3 (4.62)	5 (5.88)	2 (3.08)	0 (0.00)	10 (3.31)	
GIST	3 (6.25)	0 (0.00)	1 (1.18)	0 (0.00)	1 (2.56)	5 (1.66)	
HCC	0 (0.00)	0 (0.00)	13 (15.29)	0 (0.00)	0 (0.00)	13 (4.30)	
Cholangiocellular carcinoma	0 (0.00)	1 (1.54)	7 (8.24)	0 (0.00)	0 (0.00)	8 (2.65)	
IPMN	0 (0.00)	9 (13.85)	0 (0.00)	0 (0.00)	0 (0.00)	9 (2.98)	
Sarcoma	0 (0.00)	1 (1.54)	3 (3.53)	0 (0.00)	5 (12.82)	9 (2.98)	
Adenoma	0 (0.00)	2 (3.08)	2 (2.35)	2 (3.08)	2 (5.13)	8 (2.65)	
Benign cyst	0 (0.00)	1 (1.54)	1 (1.18)	0 (0.00)	1 (2.56)	3 (0.99)	
Other	3 (6.25)	8 (12.31)	9 (10.59)	3 (4.62)	13 (33.33)	36 (11.92)	
T stage^b^							
T0	1 (2.27)	0 (0.00)	1 (1.25)	0 (0.00)	0 (0.00)	2 (0.69)	<0.001^a1^
T1	13 (29.55)	10 (15.63)	10 (12.50)	5 (7.69)	2 (5.71)	40 (13.89)	
T2	8 (18.18)	22 (34.38)	15 (18.75)	16 (24.62)	2 (5.71)	63 (21.88)	
T3	17 (38.64)	13 (20.31)	19 (23.75)	31 (47.69)	2 (5.71)	82 (28.47)	
T4	2 (4.55)	1 (1.56)	13 (16.25)	5 (7.69)	13 (37.14)	34 (11.81)	
TX	3 (6.82)	3 (4.69)	14 (17.50)	2 (3.08)	5 (14.29)	27 (9.38)	
Tis	0 (0.00)	0 (0.00)	0 (0.00)	3 (4.62)	0 (0.00)	3 (1.04)	
N stage^b^							
N0	18 (41.86)	16 (25.00)	22 (27.50)	42 (64.62)	9 (25.71)	107 (37.28)	<0.001^a1^
N1	6 (13.95)	15 (23.44)	18 (22.50)	9 (13.85)	7 (20.00)	55 (19.16)	
N2	10 (23.26)	15 (23.44)	9 (11.25)	10 (15.38)	2 (5.71)	46 (16.03)	
N3	4 (9.30)	0 (0.00)	0 (0.00)	0 (0.00)	1 (2.86)	5 (1.74)	
NX	5 (11.63)	3 (4.69)	23 (28.75)	1 (1.54)	5 (14.29)	37 (12.89)	
M stage^b^							
M0	27 (58.70)	32 (50.79)	23 (29.11)	42 (65.63)	10 (27.78)	134 (46.53)	<0.001^a1^
M1	7 (15.22)	7 (11.11)	38 (48.10)	13 (20.31)	9 (25.00)	74 (25.69)	
MX	12 (26.09)	9 (14.29)	10 (12.66)	6 (9.38)	6 (16.67)	43 (14.93)	
Resection margin^c^							
R0	41 (93.18)	33 (54.10)	47 (67.14)	59 (90.77)	12 (37.50)	192 (70.59)	<0.001^a1^
R1	2 (4.55)	10 (16.39)	11 (15.71)	3 (4.62)	7 (21.88)	33 (12.13)	
R2	1 (2.27)	3 (4.92)	4 (5.71)	0 (0.00)	2 (6.25)	10 (3.68)	

Data are expressed as *n* (%) unless otherwise specified

*GIST* gastrointestinal stromal tumor, *HCC* hepatocellular carcinoma, *IPMN* intraductal papillary mucinous neoplasm, *GI* gastrointestinal, *SD* standard deviation

^a^1 = Chi-square test, two-sided; 2 = ANOVA, two-sided

^b^Malignant without GIST

^c^Malignant

### Patient-Reported Symptoms and Quality of Life (Objective 1)

**Fig. 2:**
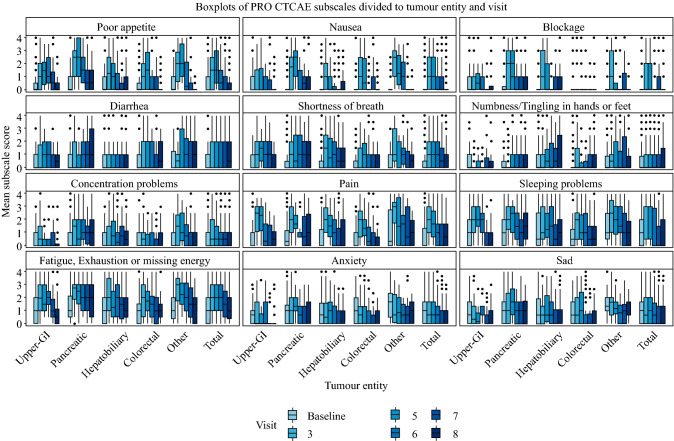
Boxplot illustrating the different symptoms of the PRO-CTCAE at each visit, divided according to tumor entity: visit 3 (3–5 postoperative days), visit 4 (6–8 postoperative days), visit 5 (at discharge or at day 30), visit 5 (3 months postoperatively), and visit 6 (6 months postoperatively). *PRO-CTCAE* patient-reported outcomes version of the Common Terminology Criteria for Adverse Events

**Fig. 3:**
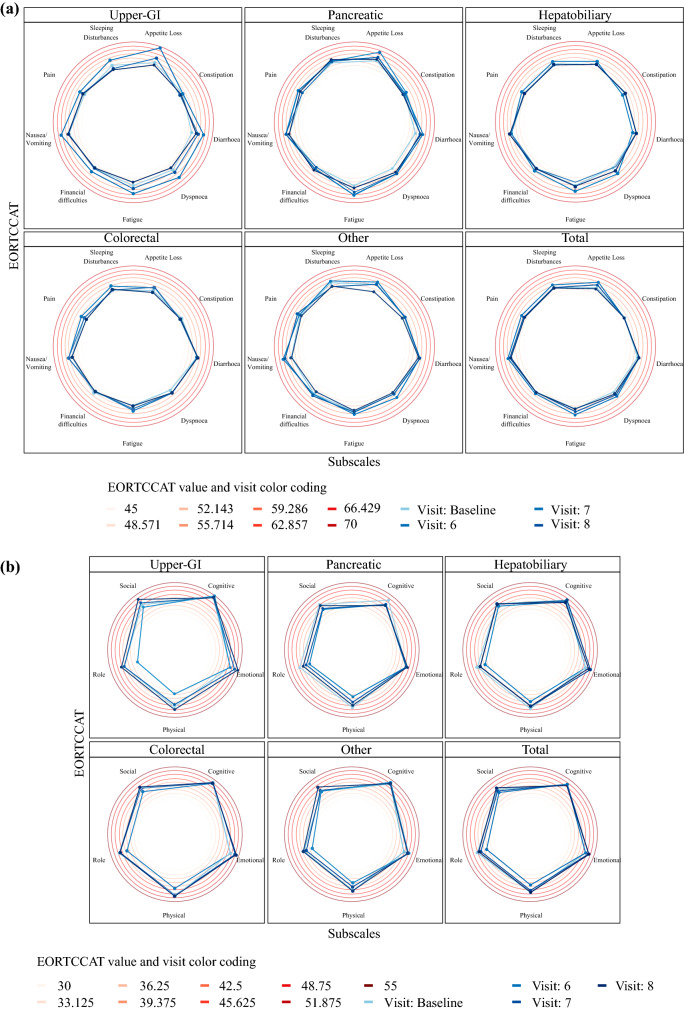
**a** Symptom scale illustrating the different subscales of the CAT EORTC QLQ-C30 at each visit according to tumor entity. **b** Functional scale illustrating the different subscales of the CAT EORTC QLQ-C30 at each visit divided according to tumor entity. *CAT EORTC QLQ-C30* computerized adaptive testing version of the EORTC health-related quality of life tool, *EORTC* European Organisation for Research and Treatment of Cancer

PRO measures and HRQoL for the separate tumor entities and the entire cohort are depicted in Figs. 2 and 3. The PRO-CTCATE symptoms (Fig. 2) ‘poor appetite’, ‘fatigue’, ‘exhaustion or missing energy’, and ‘sleeping problems’ increased postoperatively, climaxed between POD 10–30 (visit 5 or 6), and decreased 3–6 months after surgery (visit 7 and 8). In contrast, ‘diarrhea’ increased postoperatively and remained constant over time. ‘Nausea’ was significantly increased at visits 3, 5, and 6 compared with baseline, but normalized 3 months (visit 7) after surgery. Similarly, ‘anxiety’ only decreased during long-term follow-up (3 and 6 months after surgery; visits 7 and 8). Two weeks after surgery (visit 5), elevated levels were measured for ‘fatigue’ and ‘exhaustion or missing energy’. Even 1 month after surgery (visit 6) patients stated ‘concentration problems’ significantly more often than before surgery. Six months after surgery (visit 6) no PRO-CTCAE™ symptoms differed significantly compared with baseline, except ‘diarrhea’.

The CAT EORTC HRQoL questionnaire (Fig. 3) revealed that patients at visit 8 were more often confronted with ‘dyspnea’ and ‘financial difficulties’ compared with baseline (*p* = 0.032); however, patients stated higher ‘social functioning’ at this timepoint (*p* = 0.021) compared with the preoperative level. Overall HRQoL scores differed significantly between baseline and visits 6, 7, or 8 (*p* < 0.05).

### Perioperative Complications and Quality of Life (Objectives 2 and 3)

Of 303 patients, 302 could be analyzed regarding postoperative complications; 112 patients had no complications, 86 patients had minor complications (grade II), and 104 patients had major complications (grade III–IV) according to the DCC. All PRO-CTCAE™ symptoms correlated only (very) weakly with complications (minor or major) (Fig. 3a). Looking at major complications, the PRO-CTCAE™ symptoms of ‘constipation’ (blockage) at visit 3 (POD 3–5; *p* = 0.027) and ‘poor appetite’ at visit 6 (1-month postoperatively; *p* = 0.002) were significantly correlated with major complications.

Similarly, CAT EORTC measures exhibited only weak correlations with overall postoperative morbidity (Fig. 3b). Three months after surgery ‘financial difficulties’ were significantly correlated to major complications (*p* = 0.027). Correlations were overall weak, and there was no moderate (0.40–0.59) or strong (>0.60) correlation between short-term clinical outcomes (complications) and any PRO-CTCAE™ or CAT EORTC items (Fig. 3b).

### Overall Survival in Relation to Patient-Reported Outcomes (Objective 4)

In most scales, there was no correlation between overall survival and PRO-CTCAE™ symptoms. Only the PRO-CTCAE™ symptom ‘poor appetite’ at baseline (hazard ratio [HR] 1.530, 95% confidence interval [CI] 1.052–2.223; *p* = 0.0259), as well as a higher reported level of ‘sadness’ at visit 3, significantly increased the risk of death (HR 1.689, 95% CI 1.092–2.612; *p* = 0.019). Three months after surgery (visit 7) the symptoms of ‘poor appetite’ (HR 3.973, 95% CI 1.180–13.373; *p* = 0.0259), ‘nausea’ (HR 4.578, 95% CI 1.105–18.966; *p* = 0.0360), and ‘concentration problems’ (HR 4.268, 95% CI 1.115–16.339; *p* = 0.0341) significantly increased the risk of death.

Similarly, for the CAT EORTC QLQ-C30 3 months after surgery (visit 7), higher scores in ‘nausea/vomiting’ (HR 1.109, 95% CI 1.026–1.198; *p* = 0.0088) and ‘pain’ (HR 1.298, 95% CI 1.014–1.660; *p* = 0.0382) correlated with an increased risk of death. On the contrary, higher CAT EORTC QLQ-C30 scores in ‘role function’ (HR 0.819, 95% CI 0.674–0.996; *p* = 0.045) and ‘quality of life’ (HR 0.881, 95% CI 0.779–0.997; *p* = 0.0447) at 3 months after surgery correlated with lower risk of death within the study period.

### Feasibility of Student-Led Clinical Research (Objectives 5 and 6)

PRO data completion at baseline was, on average, 88.1 ± 0.5% for CAT EORTC and 91.7 ± 0.2% for PRO-CTCAE™, with little variance between trial sites (electronic supplementary Fig. 3). PRO CTCAE data completeness dropped below 90% at visit 3 (on average, 76.2%). Data completeness for PRO measures during follow-up after 1, 3, and 6 months was 42.6 ± 0.2%, 58 ± 0.3% and 55.5 ± 0.4% for PRO-CTCAE™, respectively, and 38.8 ± 0.4%, 56.5 ± 0.4% and 57.6 ± 0.3% for CAT EORTC, respectively (electronic supplementary Fig. 3).

In total, 37 hospitals were contacted by the CHIR-*Net* SIGMA study group (electronic supplementary Fig. 2). Of 37 contacted sites, 15 trial sites were unable to participate because no students and/or supervising surgeons were able to form joint teams within the recruitment period. The remaining 22 sites initiated the study, however 7 sites dropped out due to problems regarding trial infrastructure or approval from local Ethics Committees, resulting in 15 of 22 initiated sites (68.18%) enrolling patients in the study.

### Further Analysis

Overall survival was analyzed via Kaplan–Meier graphs (electronic supplementary Fig. 4) and showed significant differences between the tumor entities.

## Discussion

**Fig. 4:**
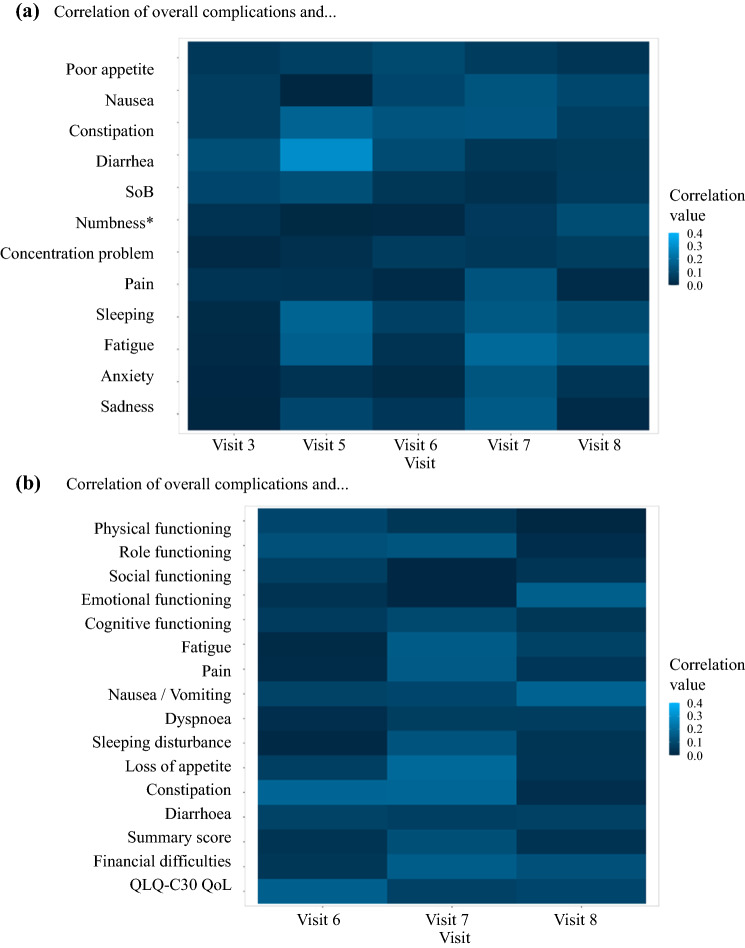
Correlation of overall postoperative complications with **a** PRO-CTCAE symptom scores and **b** CAT EORTC QLQ-C30 items. *QoL* quality of life, *PRO-CTCAE* patient-reported outcomes version of the Common Terminology Criteria for Adverse Events, *CAT EORTC QLQ-C30* computerized adaptive testing version of the EORTC health-related quality of life tool, *EORTC* European Organisation for Research and Treatment of Cancer, *SoB* shortness of breath

PATRONUS was the first multicenter, student-led clinical study in Germany. It combined proof-of-feasibility of student-led clinical research on the one hand and evaluation of clinical research questions on the other hand. As study conception, planning, acquisition, and analysis of data were performed by more than 100 medical students at 15 sites across Germany under the supervision of academic surgeons, the study is a model for research-based learning, which is a concept that refers to a trend in higher education, namely to provide students with the opportunity to gain knowledge by conducting their own scientific inquiries or investigations that are of interest to the scientific or medical community.^26^ More than 60% of the centers that were initiated finally enrolled patients (Fig. 4), showing the widespread acceptance and feasibility of this concept.

Several findings are noteworthy. First, measurements of cancer-associated symptoms via the newly developed PRO-CTCAE™, as well as assessment of HRQoL via the new CAT version of the EORTC QLQ-C30, were technically possible via an electronic data capture system. Data completeness was >90% at baseline and >80% in the immediate postoperative period (POD 3–5), therefore we concluded that PRO measurement was accepted by patients undergoing major abdominal surgery. The two new PRO tools are attractive for a wider application in surgical oncology as they allow a standardized symptom assessment across multiple domains of cancer treatment, as both tools have been employed in medical oncology and palliative care.^16^,^27^,^28^ They would therefore allow assessment of the total cancer and treatment burden from the perspective of individual patients or patient groups along an entire healthcare pathway.^29^ As both PRO measures can be tailored to specific needs and situations, either by offering a wide set of standardized symptom scores or by using a CAT approach, these tools allow a more personalized PRO assessment, thus alleviating the burden of answering improper, lengthy, standard questionnaires.^7^,^30^ This also elucidates one of the limitations of our study. In order to catch PRO symptoms across multiple cancer types, we used cancer-specific, but not disease-specific, PRO measurements, i.e. the CAT version of the EORTC QLQ-C30 and a general set of 12 cancer symptoms as recommended by the NCI.^14^ Therefore, depending on cancer type and intervention, patients might additionally consider other items important that have not been covered with our set of measures. Future studies using disease-specific PRO measures will need to fill this gap.

To our knowledge this is the first report of PRO-CTCAE™ and CAR EORTC QLQ-C30 data in surgical oncology. The data provided in this publication and its supplements can thus be used for sample size calculation in future trials or for standardization and quality measurements in regular care. Although symptoms are directly affected by oncological surgery postoperatively (Fig. 2a), overall HRQoL, as well as functional subscales of the CAT EORTC QLQ-C30, either normalized or even improved compared with baseline within 6 months after surgery. This has been previously reported for other PRO measures^31^,^32^ and confirms the multidimensional construct of HRQoL, which extends beyond symptom assessment and can be stable even under severely adverse conditions.^33^ Overall, these results confirm major abdominal surgery as an adequate intervention from the patient’s HRQoL point of view.

There was a lack of moderate or strong correlation between PRO measures and postoperative morbidity. Consequently, we see a challenge for the use of patient-reported core cancer symptoms in predicting postoperative complications. Although this has been reported in other trials using different symptom and HRQoL measures,^33^ other studies have reported the opposite.^34^ In addition, Dumitra et al. reported that correlations also depend on the complication grading system and not only on the complication itself.^35^ Therefore, future studies with larger sample sizes will need to elucidate this association more clearly. Given the small sample size in some of the subgroups, larger cohorts are needed to identify specific PRO symptoms that can be used as early detection markers for looming surgical complications. To this end, disease-specific PRO measures and symptom scores could be used for specific tumor types. The idea of using automatized symptom reporting in conjunction with clinical examination and laboratory findings to predict postoperative complications is attractive in surgical oncology, especially in settings where major complications are frequent.

There are several limitations to our study. First, although 15 sites enrolled 303 patients in the PATRONUS study (average 20.2 patients per site), we fell short of the intended 30 patients per site. Furthermore, we were unable to recruit the planned 30 trial sites, mostly because of delays in patient recruitment due to the lengthy process of obtaining positive ethic votes for each individual site. During this time, some mini-teams consisting of students and academic surgeons broke apart. Furthermore, it needs to be pointed out that not all patients underwent resection and that a small subgroup of patients had benign histologies on their final pathology report (Table 2). We kept these patients in the final analyses in line with our prespecified inclusion criteria (preoperative confirmed or suspected malignancy). Another shortcoming is that average data completeness of PRO measures dropped to 55–58% after 3 and 6 months postoperatively. A recent systematic review of US FDA cancer trials reported a median PRO data completion rate of 89%, ranging from 33 to 100%.^36^ Reasons for the drop in data completeness during follow-up was the relatively long follow-up period, which put a considerable strain on the already busy schedule of most medical students. Consequently, other successful student-led studies in the UK focused on shorter data capture periods or were cross-sectional audits rather than prospective studies.^37^–^39^ Data completeness might be increased by strengthening the centralized automated monitoring via the electronic capture system. This would give immediate feedback and would avoid a delayed query process. In addition, query management and response need to be part of the pre-study training workshop.^20^

In addition, most participating hospitals were large tertiary university centers, which limits the external validity of our results. Therefore, patient groups and surgeries performed (Tables 1, 2) might not reflect surgical practice in other hospitals. Finally, although intended to increase knowledge, skills, and competencies in clinical research of participating medical students, we did not measure educational goals in the current study. However, evaluation of our clinical investigator training prior to study participation showed an increase in clinical research knowledge in a pre/post test.^20^ Several studies have shown that exposure to research during medical school correlates with engagement in research later on.^40^,^41^

## Conclusion

Despite the fact that only low correlations between patient-reported symptoms and complications were found, PRO-CTCAE™ and CAT EORTC QLQ-C30 are promising PRO tools for surgical oncology as they elucidate the patients’ perspective on surgical treatment and can be implemented electronically in the postoperative setting and after discharge. Furthermore, patients undergoing major abdominal surgery exhibit HRQoL scores similar or better than preoperatively. Student-led multicenter clinical research is feasible. Currently, the CHIR-*Net* SIGMA study group is conducting a randomized controlled trial investigating the effect of fitness tracker and enhanced postoperative mobilization on postoperative complications.^42^ This trial (EXPELLIARMUS; UTN: U1111-1228-3320) takes into account the lessons learned from PATRONUS.

### Supplementary Information

Below is the link to the electronic supplementary material.

Supplementary material 1

Supplementary material 2

Supplementary material 3

Supplementary material 4

## Appendix: List of collaborators

Coordinating Investigator

Mihaljevic, André, Mihaljevic@uni-heidelberg.de

Steering Committee

*National Leads*

Fink, Christopher, Christoph-Fink@gmx.de

Frey, Pia, pi_frey@yahoo.de

Friedrich, Mirco, friedrich.mirco@googlemail.com

Leuck, Alexander, leuck@stud.uni-heidelberg.de

Rädeker, Lukas, lukas@sigma-studies.org

Schwab, Marius, marius.schwab@stud.uni-heidelberg.de

*Project Management*

Doerr-Harim, Colette, Colette.Doerr-Harim@med.uni-heidelberg.de

Tenckhoff, Solveig, solveig.tenckhoff@med.uni-heidelberg.de

*Statistician*

Feißt, Manuel, feisst@imbi.uni-heidelberg.de

Writing Committee

Mihaljevic, André, Andre.Mihaljevic@med.uni-heidelberg.de

Tenckhoff, Solveig, solveig.tenckhoff@med.uni-heidelberg.de

Schwab, Marius, marius.schwab@stud.uni-heidelberg.de

Doerr-Harim, Colette, Colette.Doerr-Harim@med.uni-heidelberg.de

Feißt, Manuel, feisst@imbi.uni-heidelberg.de

Faqar-Uz-Zaman, Sara Fatima, SaraFatima.Faqar-Uz-Zaman@kgu.de

Brindl, Niall, brindl@stud.uni-heidelberg.de

Tu, Thomas Jing Zhi, tu@stud.uni-heidelberg.de

Studier-Fischer, Alexander, alexander@studier-fischer.de

Kuner, Charlotte, kuner@stud.uni-heidelberg.de

Gsenger, Julia, gsenger@stud.uni-heidelberg.de

Berlin

*Local Lead*

Möhwald, Clara, clara.moehwald@charite.de

Prem, Markus, Markus.Prem@charite.de

*Mini-Team Members*

Von der Waydbrink, Henriette, henriette.von-der-waydbrink@charite.de

Tielking, Katharina, katharina.tielking@charite.de

Rahbari, Mohammad, mohammad.rahbari@umm.de

*Surgical Facilitator*

Bertram, Boortz, bertram.boortz@charite.de

Neudecker, Jens, Jens.Neudecker@charite.de

Dresden

*Local Lead*

Jörke, Linda, linda.joerke@gmail.com

*Mini Team Members*

Findeisen, Lisa, lifi1201@gmail.com

Fichtel, Pascal, pascal.fichtel@gmail.com

Bigge, Richard, richard.bigge@me.com

*Surgical Facilitator*

Nuh, Rahbari, Nuh.Rahbari@uniklinikum-dresden.de

Bork, Ulrich, Ulrich.Bork@uniklinikum-dresden.de

Frankfurt am Main

*Local Lead*

Faqar-Uz-Zaman, Sara Fatima, SaraFatima.Faqar-Uz-Zaman@kgu.de

*Mini-Team Members*

Klein, Adele, adeleklein@ymail.com

Stojanovich, Strahin, strahinjastojanovich@hotmail.com

*Surgical Facilitator*

Schnitzbauer, Andreas A., Andreas.Schnitzbauer@kgu.de

Freiburg

*Local Lead*

Vorbach, Samuel, Samuel.Vorbach@t-oNational Leadine.de

*Mini Team Members*

Braune, Jan, jan.braune@posteo.de

Scholz, Alexander, alexander-scholz@oNational Leadine.de

Kreifels, Paul, pkreifels@outlook.de

Zell, Daniel, daniel.zell@gmx.de

Van de Lo, Tim, tivandeloo@aol.com

*Surgical Facilitator*

Holzner, Philipp, philipp.holzner@uniklinik-freiburg.de

Martini, Verena, verena.martini@uniklinik-freiburg.de

Hamburg

*Local Lead*

Hinz, Nico, hinz_nico@gmx.de

*Mini Team Members*

Finger, Anna Ekaterina, 7075905@stud.uke.uni-Hamburg.de

Kokaly, Nadia, nadia.kokaly@stud.uke.uni-hamburg.de

Kosch, Ricardo, ricardo.kosch@stud.uke.uni-hamburg.de

Piel, Sharlaine Yun Lan, sharlaine.piel@stud.uke.uni-hamburg.de

*Surgical Facilitator*

Tachezy, Michael, m.tachezy@uke.de

Heidelberg

*Local Lead*

Gablenz, Eva, e.vongablenz@stud.uni-heidelberg.de

Alhalabi, Obada T., obadath@hotmail.com

Sliwinski, Svenja, svenja.sliwinski@hotmail.de

*Mini-Team*

Marx, Alexander, a.marx95@gmx.de

AuYeung, Annette, annette_a@rocketmail.com

Achilles, Christina, christina-achilles@t-oNational Leadine.de

Betge, Fynn, fynn.betge@gmx.de

Kühn, Jana, jana-f-kuehn@web.de

Al Halabi, Karam, karam.alhalabi@hotmail.com

von Fritsch-Seerhausen, Lennart, L.Fritsch-Seerhausen@stud.uni-heidelberg.de

Beck, Lydia, lydia.beck@stud.uni-heidelberg.de

Truant, Marc, Truant@stud.uni-Heidelberg.de

Preuß, Sophie, sophie.preuss@gmx.de

Waider, Stella, Waider@stud.uni-heidelberg.de

Seiboldt, Till, seiboldt@stud.uni-heidelberg.de

Brindl, Niall, brindl@stud.uni-heidelberg.de

Tu, Thomas Jing Zhi, tu@stud.uni-heidelberg.de

*Surgical Facilitator*

Mihaljevic, André, Mihaljevic@uni-heidelberg.de

Heidelberg, Institute of Medical Biometry and Informatics

Sander, Anja, sander@imbi.uni-heidelberg.de

Honecker, Hannah, Hannah.Honecker@gmx.de

Seide, Svenja, seide@imbi.uni-heidelberg.de

Weber, Dorothea, weber@imbi.uni-heidelberg.de

Büsch, Christopher, buesch@imbi.uni-heidelberg.de

Herne

*Local Lead*

Wagner, Florian, florian.wagner-l6z@ruhr-uni-bochum.de

*Mini-Team*

Reckmann, Lisa, lisa.reckmann@web.de

Sonnenberg, Anna-Maria, sonnenberg.anna@gmail.com

*Surgical Facilitator*

Zaczeck, Michael, michael.zaczek@junge-chirurgie.de

Kiel

*Local Lead*

Buech, Joscha, joscha.buech@med.uni-muenchen.de

*Mini-Team*

Luden, Laura

Autenrieb, Marie-Pauline, stu211487@mail.uni-kiel.de

*Surgical Facilitator*

Taivankhuu, Terbish, Terbish.Taivankhuu@uksh.de

Langen

*Local Lead*

Tröster, Desiree, desiree.troester@gmail.com

*Mini-Team*

Engster, Simon, simon.engster@gmail.com

*Surgical Facilitator*

Buia, Alexander, a.buia@t-oNational Leadine.de

Prof. Dr. Hanisch, Ernst

Müller-Debus, Charlotte, CharlotteFriederieke.Mueller-Debus@uksh.de

Mainz

*Local Lead*

Heinrich, Alexander, ali.heinrich@gmx.de

Rasel, Hannah, hannah.rasel@web.de

*Surgical Facilitator*

Nergiz, Kartal, gnk2706@web.de

Prof. Dr. med. Lang, Hauke, Hauke.lang@unimedizin-mainz.de

Watzka, Felix, Felix.watzka@unimedizin-mainz.de

Mannheim

*Local Lead*

Pohl, Laura, Laura.pohl@stud.uni-heidelberg.de

Gsenger, Julia, gsenger@stud.uni-heidelberg.de

*Mini-Team*

Makabe, Akiko Lucia, akiko@makabe.de

Seckler, Anna-Maria, Seckler@stud.uni-heidelberg.de

Misiraj, Blerina, blerina_misiraj@hotmail.de

Kerem, Carmel, kerem@stud.uni-heidelberg.de

Lull, Christian, Lull@stud.uni-heidelberg.de

Voigt, Jonas, jonas_voigt@me.com

Beier, Julian Pascal, Julian.Beier@stud.uni-heidelberg.de

Schütze, Leon, schuetze.leon@gmail.com

Dreyer, Leonhard, Leonhard.dreyer@stud.uni-Heidelberg.de

Kleinsorge, Marie Therese, marie.kleinsorge@gmx.de

Nguyen, Thao, thao.tnguyen98@gmail.com

Thäwel, Thomas, thomas.thaewel@gmail.com

Günther, Tobias, usbler@googlemail.com

*Surgical Facilitator*

Herrle, Florian, Florian.herrle@umm.de

München LMU

*Local Lead*

Sadeghi, Diana, diana.sadeghi@gmx.at

*Mini-Team*

Scheuerpflug, Anne, anne.scheuerpflug@web.de

Apfelbeck, Antonia, antonia.apfelbeck@web.de

Schömer Cuenca, Sebastian, sebastian.schoemer@gmail.com

*Surgical Facilitator*

Albertsmeier, Markus, markus.albertsmeier@med.uni-muenchen.de

Zimmermann, Petra, Petra.Zimmermann@med.uni-muenchen.de

von Ehrlich-Treuenstätt, Viktor, viktor.vehrlich@med.uni-muenchen.de

München TU

*Local Lead*

Kopp, Alessia, alessia.v.kopp@gmail.com

Schmitzer, Lena, lenaschmitzer@web.de

*Surgical Facilitator*

Reim, Daniel, daniel.reim@tum.de

Schirren, Rebekka, rebekka.schirren@tum.de
