# Supplementary figures and images for: Multicenter Prospective Cohort Study of the Patient-Reported Outcome Measures PRO-CTCAE and CAT EORTC QLQ-C30 in Major Abdominal Cancer Surgery (PATRONUS): A Student-Initiated German Medical Audit (SIGMA) Study
Source: Ann Surg Oncol. 2021 Mar 8;28(6):3075–89. doi: 10.1245/s10434-021-09646-z (PMC8119276; doi:10.1245/s10434-021-09646-z)

## Slide 1
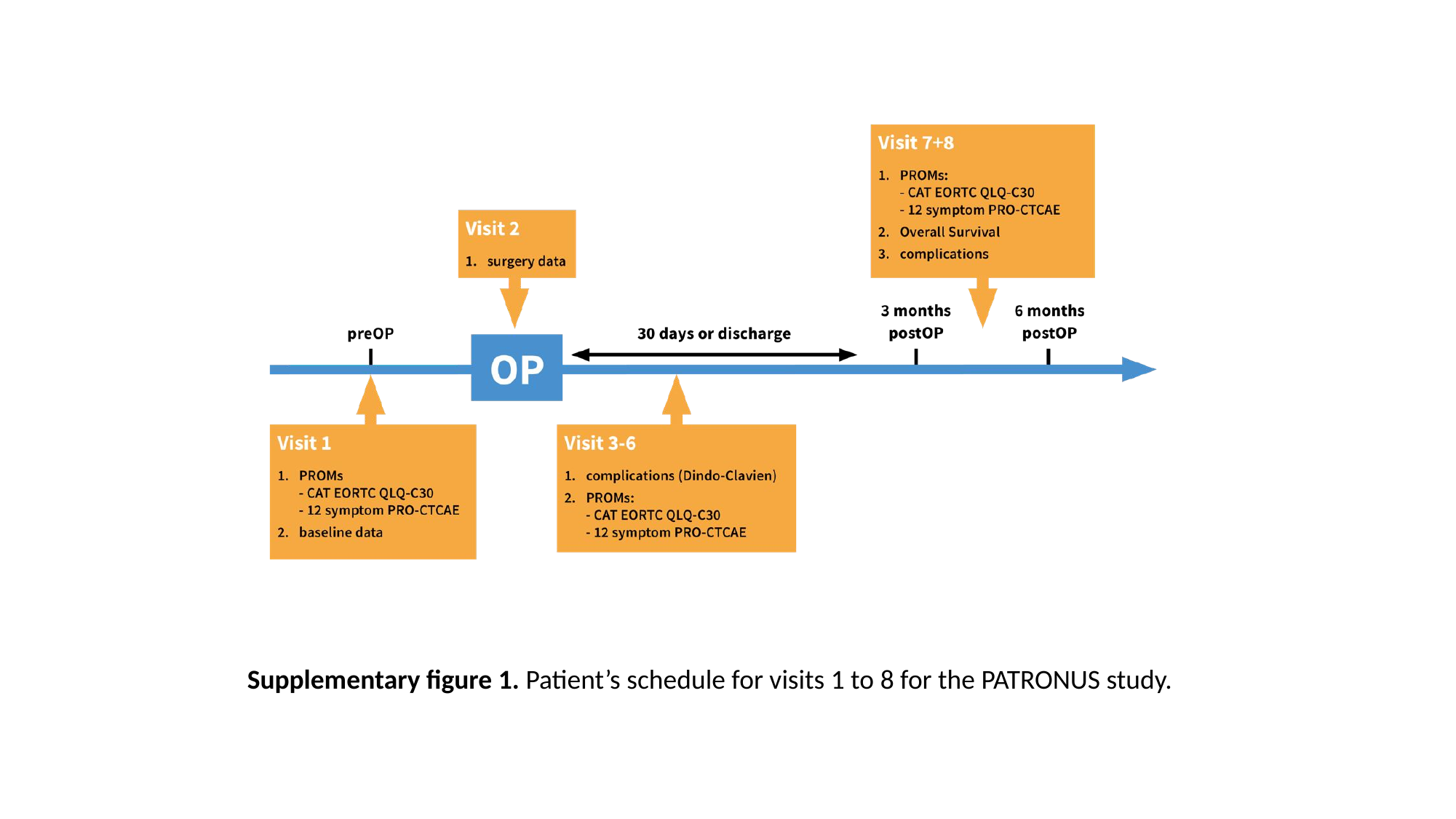

Supplementary figure 1. Patient’s schedule for visits 1 to 8 for the PATRONUS study.

Supplement: Supplementary file 1 — Supplementary material 1 [file 10434_2021_9646_MOESM1_ESM.pptx]

## Slide 1
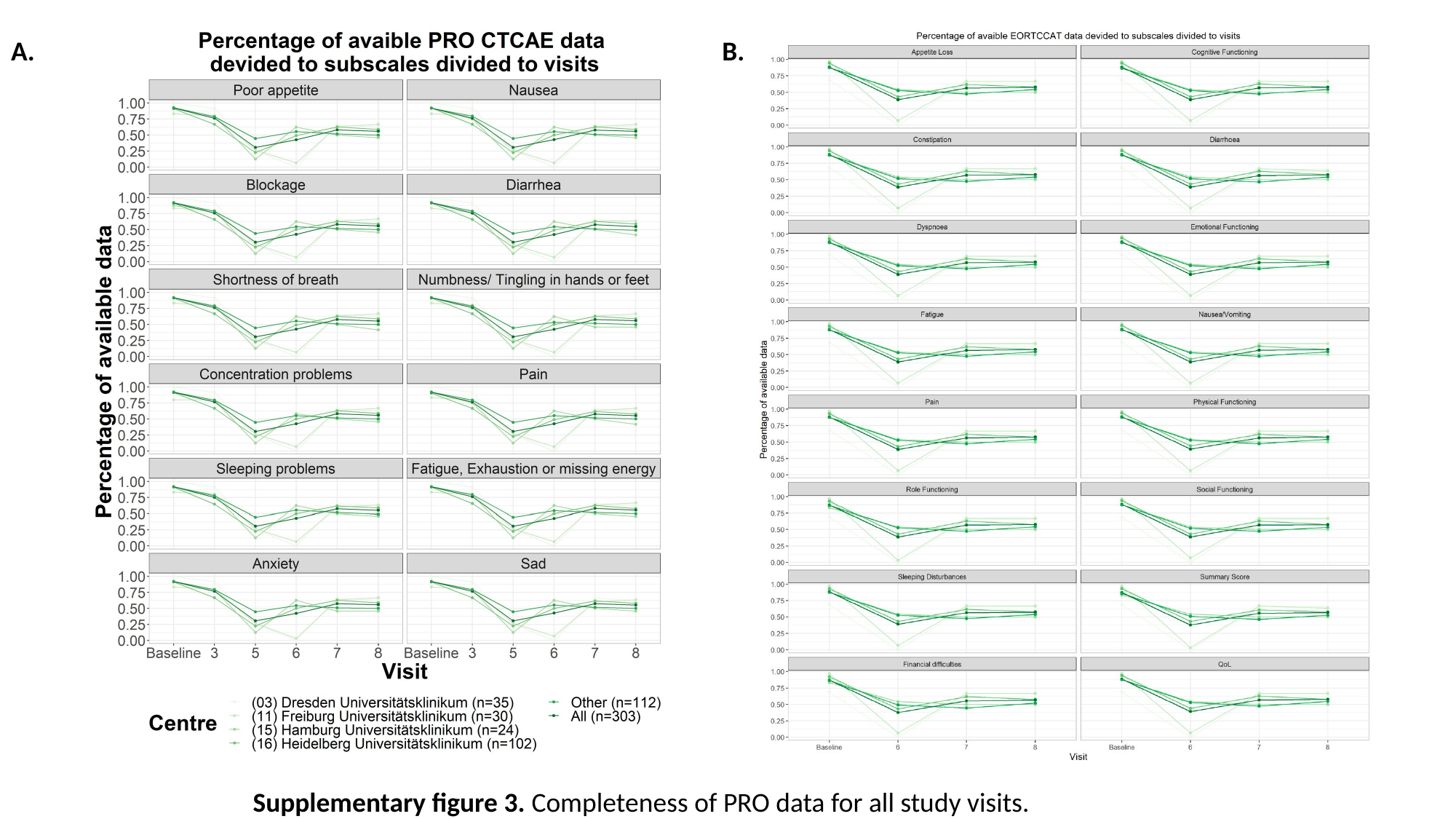

A.
B.
Supplementary figure 3. Completeness of PRO data for all study visits.

Supplement: Supplementary file 3 — Supplementary material 3 [file 10434_2021_9646_MOESM3_ESM.pptx]
