# Supplementary material for: Multicenter Prospective Cohort Study of the Patient-Reported Outcome Measures PRO-CTCAE and CAT EORTC QLQ-C30 in Major Abdominal Cancer Surgery (PATRONUS): A Student-Initiated German Medical Audit (SIGMA) Study
Source: Ann Surg Oncol. 2021 Mar 8;28(6):3075–89. doi: 10.1245/s10434-021-09646-z (PMC8119276; doi:10.1245/s10434-021-09646-z)
Supplement: Supplementary file 4 — Supplementary material 4 [file 10434_2021_9646_MOESM4_ESM.pptx]

## Slide 1
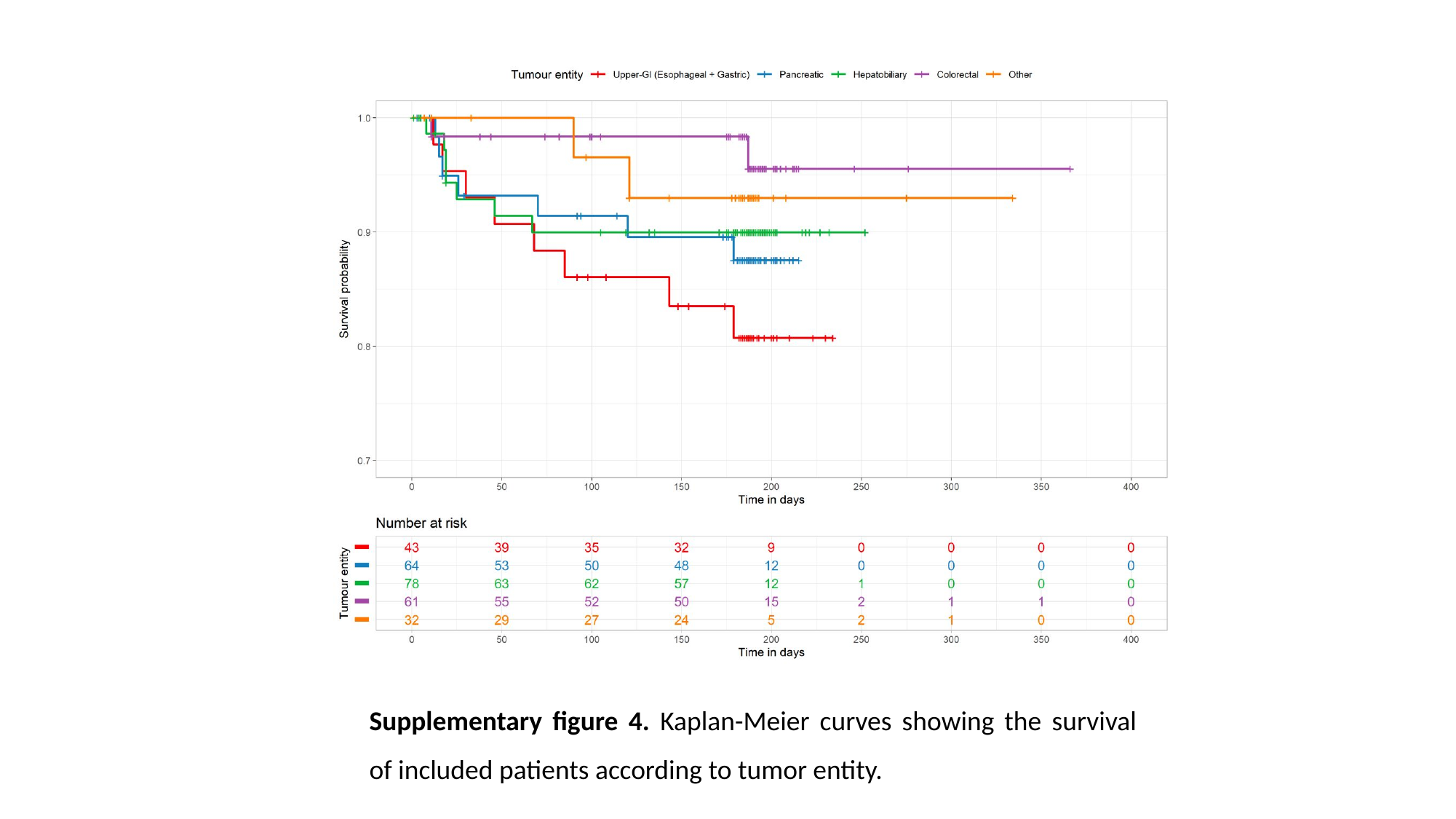

Supplementary figure 4. Kaplan-Meier curves showing the survival of included patients according to tumor entity.
